# Regulatory Design and Long-Term Growth: Insights from China’s Experience for Latin America

**DOI:** 10.12688/f1000research.178466.1

**Published:** 2026-04-02

**Authors:** Jhon Valdiglesias

**Affiliations:** 1 Center for Asian Studies, National University of San Marcos, Lima, Peru

**Keywords:** regulatory efficiency, GDP growth, China, Latin America, benchmarking

## Abstract

**Background:**

Regulatory efficiency is increasingly recognized as a key determinant of long-term economic growth. China’s institutional consolidation offers a benchmark for understanding how predictable enforcement, streamlined procedures, and coordinated governance can reduce transaction costs and foster development. In contrast, many Latin American countries face persistent administrative complexity that may hinder growth.

**Methods:**

This study applies Institutional Theory and Transaction Cost Economics to conceptualize regulatory efficiency. Using panel-data estimations with fixed- and random-effects models, the analysis examines the relationship between regulatory indicators—procedural requirements, compliance costs, and business-environment scores—and GDP per capita growth. Macroeconomic controls such as trade openness, inflation, capital formation, and rule of law are included to isolate the effect of regulatory efficiency. China serves as a benchmark case against selected Latin American countries.

**Results:**

The results show a robust and statistically significant association between lower regulatory frictions and stronger economic performance. China’s coherent and strategically aligned regulatory framework is linked to higher and more stable GDP per capita growth. In contrast, Latin America exhibits persistent procedural delays, higher compliance costs, and lower regulatory efficiency, which correspond to weaker and more volatile growth outcomes. Panel-data and dummy-variable regressions consistently confirm China’s superior performance as a reference for effective regulatory design.

**Conclusions:**

This study provides quantitative evidence that regulatory design and institutional coordination are decisive for long-term economic growth. By highlighting the growth costs of administrative inefficiency and the benefits of coherent regulation, the findings inform policy strategies aimed at improving institutional quality and fostering sustainable development. Comparative benchmarks, such as China, demonstrate how regulatory efficiency can enhance productivity and macroeconomic stability in emerging economies.

## 1. Introduction

Regulatory efficiency is increasingly recognized as a fundamental driver of economic growth and business development. Countries that maintain clear, predictable, and well-enforced regulations create an environment in which private enterprises can thrive, attracting investment and promoting innovation. China provides a striking example of such a system. Firms operating in China face minimal bureaucratic obstacles, obtain operating licenses and construction permits in just a few days, and encounter very low barriers to accessing land or other essential inputs ( Table 1). This efficiency has not only streamlined business operations but has also strengthened investor confidence and facilitated sustained economic expansion.

**Table 1.** Regulatory environment indicators for selected latin american economies.

Indicator	Peru (2023)	Chile (2010)	Colombia (2023)	Brazil (2009)	Argentina (2017)	Mexico (2023)	China (2024)
Average senior management time spent dealing with the requirements of government regulation (%)	12.3	9.9	26.2	14.2	20.5	15.8	0.8
Average days to obtain an operating license	118.3	68.5	39.3	79.7	88.2	18	6.5
Days to obtain a construction-related permit [median]	180	45	30	60	30	20	10
Percent of firms identifying business licensing and permits as a major or very severe constraint	27.8	7.5	8.5	48.4	25	16.6	0.8
Percent of firms identifying access to land as a major or very severe constraint	25.2	17	7.2	35.3	14.5	16.2	2.6

Note. Data come from the World Bank Enterprise Surveys (various years). Different years appear because only one survey year is available per country for many indicators. Japan is excluded from this descriptive table but included in the regressions. Values are for descriptive context only. Source: World Bank Enterprise Surveys, https://www.enterprisesurveys.org

By contrast, many Latin American economies continue to experience slow and fragmented regulatory processes. In countries such as Peru, Brazil, and Argentina, firms spend a considerable portion of management time navigating compliance requirements, often waiting months for operating licenses or construction permits. In addition, a substantial share of businesses perceives licensing, permits, and access to land as major constraints. These inefficiencies increase transaction costs, slow business formation, and create uncertainty that limits investment and innovation, particularly for small and medium-sized enterprises.

The differences between China and Latin America illustrate a substantial regulatory gap. While China’s system enables rapid decision-making and predictable enforcement, Latin American regulatory frameworks often generate uncertainty and operational delays. This gap raises important questions about how institutional efficiency shapes economic performance and how lessons from high-performing systems can inform policy reforms. Specifically, understanding the mechanisms by which China achieves regulatory efficiency may help Latin American countries identify strategies to simplify processes, strengthen enforcement, and reduce barriers to private sector growth.

This study aims to explore the relationship between regulatory efficiency and economic outcomes, focusing on how variations in institutional performance affect investment, competitiveness, and GDP per capita. It examines the constraints imposed by slow, fragmented regulatory processes in Latin America and contrasts these challenges with China’s streamlined, predictable system. Furthermore, it considers how elements of China’s regulatory model might be adapted to improve governance and foster a more supportive business environment in Latin American contexts.

The objectives of this research are threefold. First, it evaluates the efficiency of regulatory processes in China and selected Latin American countries, including the time required to obtain licenses and permits and the severity of perceived constraints. Second, it assesses the effects of bureaucratic fragmentation on private sector performance, investment decisions, and the ability to innovate. Third, it explores policy insights from China’s experience, highlighting opportunities to enhance regulatory frameworks in Latin America to reduce uncertainty and foster sustainable economic development.

By highlighting China as a benchmark for regulatory efficiency and comparing it to Latin American experiences, this study contributes to the literature on institutional quality and economic growth. It provides detailed evidence of how regulatory design impacts business activity, clarifies the mechanisms linking efficient regulation to higher investment and growth, and offers practical guidance for policymakers seeking to improve governance and competitiveness. The analysis demonstrates that closing the regulatory gap is not merely a technical challenge, but a critical pathway for enhancing private sector development and long-term economic performance in Latin America.

## 2. Literature review and hypothesis

### 2.1. Regulatory institutions and growth in China and Lain America

Mainstream growth theory identifies institutional quality as central to sustained development, yet the Latin American experience reveals the limits of this premise when reforms lack depth and coherence. Although dynamic panel evidence links growth to macroeconomic stability and institutional credibility ( Araujo et al., 2014), the commodity super-cycle demonstrated that much of the region’s convergence was externally driven and temporary, with structural regulatory weaknesses resurfacing once prices declined.

In contrast, China’s trajectory challenges the assumption that liberal-democratic institutions are necessary for efficiency gains: stochastic frontier estimates show significant improvements in productive efficiency relative to Brazil ( Nazmi & Revilla, 2008), achieved within a system defined by disciplined state coordination rather than liberalization. Subsequent regulatory consolidation under Xi Jinping—marked by a structural break in 2012 and reinforced anti-corruption enforcement—suggests deliberate institutional recalibration to enhance coherence and reduce rent-seeking (Valdiglesias, 2026), with measurable governance improvements associated with stronger economic performance compared to fragmented Latin American reforms ( Valdiglesias, 2025).

Sectoral evidence reinforces this asymmetry: infrastructure and electricity regulation in Latin America remain undermined by weak oversight, corruption, and limited enforcement capacity despite formal autonomy ( Suárez-Alemán et al., 2019; Ravillard et al., 2010; Andres, 2014), whereas China’s coordinated regulatory approach—even in environmental policy—demonstrates how initially restrictive measures can ultimately foster green growth and productivity spillovers ( Chen et al., 2024).

China’s expansion reinforced Latin America’s commodity dependence and revealed structural weaknesses in industrial and competition policy ( Wise & Quiliconi, 2007; Wise, 2016). Although often framed as liberalizing reforms ( Hamner, 2002), China’s model remains anchored in state coordination through SOEs and strategic planning ( Hearn and León-Manríquez, 2011), diverging sharply from Latin America’s fragmented post-2009 regulatory adjustments. Ongoing deficiencies in corporate governance, investor protection, SME finance, and enforcement capacity ( Chong and Lopez de Silanes, 2007; Valdiglesias, 2012; Cardoza et al., 2016) further contrast with the centralized coordination and transaction-cost efficiency observed in East Asia ( OECD Development Centre, 2015).

Empirical evidence reinforces this divergence. Independent regulatory agencies with credible enforcement powers are associated with higher investment and improved infrastructure outcomes ( Gutierrez and Berg, 2000). East Asian—particularly Chinese—firms operating in Latin America, such as in Peru’s mining sector, benefit from alignment between host-country openness and home-country strategic coordination ( Moran et al., 2012). At the same time, East Asian investment abroad often prioritizes pragmatic market access over strict adherence to global governance norms ( Dollar, 2017), highlighting a selective application of regulatory discipline. Domestically, East Asian private firms have historically navigated weak property rights and shifting policy landscapes through dense state-business networks ( Ahlstrom et al., 2008; Valdiglesias, 2018), underscoring that regulatory efficiency in the region is neither purely liberal nor institutionally neutral.

China’s expanding presence in Latin America illustrates how regulation can function as an instrument of strategic statecraft. Policy banks, bilateral agreements, and coordinated investment frameworks reinforce regulatory alignment with national development objectives ( Fornes and Mendez, 2018; Jenkins, 2022). Although private governance mechanisms increasingly complement formal rules ( Mayer and Gereffi, 2010), their effectiveness ultimately depends on enforcement capacity—an area where many Latin American countries remain deficient. Historical privatization experiences further reveal that liberalization alone does not guarantee efficiency gains when regulatory oversight is weak or fragmented ( Chong and Lopez de Silanes 2005).

Foreign direct investment from East Asia generates ambivalent outcomes in Latin America. While contributing to employment, structural change, and technological transfer ( Girón, 2025; Rocha, 2025; Freites, 2024; Zambrano-Monserrate, 2025; Mazé et al., 2024), it also operates within regulatory environments characterized by limited monitoring and enforcement ( Lopez and Munoz, 2020; De Barrios et al., 2023; Albright, 2025; Vieira et al., 2023). Allegations of labor and environmental violations ( Irwin and Gallagher, 2013) should therefore be understood within systemic regulatory weaknesses rather than attributed solely to foreign actors. More broadly, institutional fragility tends to deter sustained productive investment, raise transaction costs, and constrain SME expansion ( Chong and Lopez de Silanes, 2007; Valdiglesias, 2024; McKay et al., 2018; Vassolo et al., 2012).

Recent scholarship emphasizes that East Asia’s regulatory advantage lies in the integration of industrial policy, pilot free trade zones, coordinated oversight, and infrastructural investment ( Shen et al., 2025; Sims et al., 2025). Stronger governance coherence and policy integration reduce uncertainty and facilitate long-term planning ( Kastner and Pearson, 2021; Kim and Dong, 2025; Weng and Li, 2024; Zhang et al., 2022). In contrast, Latin America’s fragmented enforcement structures, informal practices, and uneven institutional quality undermine predictability and corporate decision-making ( Walsh and Ferro, 2020; Stallings, 2024; Gaganis et al., 2021; Reyes, 2021; Mechelli and Cimini, 2020). Even reforms in state-owned enterprises have delivered only partial efficiency gains without resolving systemic governance constraints ( Bello et al., 2023; Križić, 2021; Abreo et al., 2021).

Infrastructure and digital integration further accentuate the gap. East Asia’s coordinated investments in logistics, transportation, and digital systems enhance regulatory effectiveness and lower operational costs, whereas Latin America’s fragmented frameworks limit the developmental impact of FDI ( Soto and Martinez-Cobas, 2024; Lei, 2024; Urrego-Sandoval and Pardo, 2023; Lopez and Munoz, 2020; Kastner and Pearson, 2021). Informal institutions and public perceptions also shape compliance and reputational risk differently across regions, reinforcing regulatory outcomes in East Asia while amplifying volatility in Latin America ( Gaganis et al., 2021; Rousham et al., 2023; Mihut et al., 2025; Bezuidenhout et al., 2021; Wang and Feng, 2023; Albright, 2025; Zhou, 2023).

Importantly, methodological differences in the literature mirror these institutional asymmetries. Research on East Asia frequently employs longitudinal and quantitative approaches capable of identifying causal relationships between regulation and firm-level performance, whereas studies on Latin America remain more descriptive and fragmented, limiting systematic evaluation of welfare effects ( Vrontis et al., 2024; Rocha, 2025; Kim and Dong, 2025; Mechelli and Cimini, 2020; Reyes, 2021; Stallings, 2024).

Taken together, the literature suggests that China—and East Asia more broadly—offers not a replicable blueprint but a benchmark illustrating how regulatory efficiency emerges from coherent governance, strategic coordination, and credible enforcement. For Latin America, strengthening institutional capacity, ensuring consistent rule application, and preventing regulatory capture are prerequisites for translating reform into sustained economic growth ( Vorotnikova, 2025; Bello et al., 2023; The World Bank, 2025). The central analytical question, therefore, is not whether Latin America can imitate East Asia, but how regulatory efficiency—understood as predictable, enforceable, and strategically aligned governance—can be constructed within its distinct institutional constraints.

### 2.2. Theoretical framework and hypothesis

Institutional Theory provides the analytical foundation for understanding how regulatory structures shape economic outcomes. Economic performance does not emerge solely from market forces, but from the configuration of formal rules—laws, regulatory agencies, enforcement mechanisms—and informal norms that stabilize expectations and structure incentives ( North, 1990; Scott, 2008). Institutions reduce uncertainty by making behavior predictable; when they fail to do so, economic actors face higher risks, shorter planning horizons, and lower incentives to invest. In developing economies, where enforcement is often uneven, informal arrangements may temporarily compensate for weak formal governance. However, such compensatory mechanisms rarely substitute for credible regulation; instead, they tend to entrench opacity, increase discretion, and elevate transaction costs. Latin America exemplifies this institutional dualism: regulatory instability, politicized oversight, and fragmented enforcement undermine predictability, discouraging long-term capital formation and innovation.

New Institutional Economics (NIE) strengthens this argument by explicitly linking institutional quality to efficiency and welfare outcomes ( Williamson, 1975, 1985). Institutions determine the cost structure of economic exchange. When regulatory frameworks are coherent and consistently enforced, they lower negotiation, monitoring, and compliance costs, facilitating productive investment. Conversely, weak or inconsistently applied rules raise the cost of contracting, expand opportunities for rent-seeking, and distort resource allocation. Transaction Cost Economics (TCE) sharpens this logic by conceptualizing firms as adaptive responses to institutional constraints ( Williamson, 1979, 1985). In environments characterized by regulatory unpredictability, firms internalize uncertainty through vertical integration, informal arrangements, or strategic non-compliance. While such strategies may ensure survival, they reduce aggregate efficiency and constrain productivity growth.

Within this framework, regulatory efficiency is not synonymous with deregulation. Rather, it refers to the capacity of the state to design clear rules, enforce them credibly, and coordinate policy objectives across sectors. Efficient regulation minimizes administrative burdens without sacrificing oversight, reduces discretionary enforcement, and aligns incentives between public authorities and private actors. The critical variable, therefore, is not the size of the state but its organizational coherence and enforcement credibility.

China represents a benchmark case of regulatory consolidation within a state-led model. Its institutional architecture integrates bureaucratic discipline, strategic coordination, and policy continuity, reducing uncertainty and transaction costs despite limited political liberalization. Regulatory predictability—particularly in licensing, property formalization, and industrial policy—creates stable expectations that support investment and long-term planning. In contrast, many Latin American economies exhibit fragmented regulatory regimes in which formal reforms coexist with weak enforcement and politicized implementation. This disjunction generates higher compliance costs, regulatory volatility, and diminished investor confidence, ultimately constraining productivity and per capita growth.

Building on these theoretical foundations, this study advances the proposition that regulatory efficiency—understood as institutional coherence, credible enforcement, and reduced transaction costs—constitutes a decisive determinant of economic performance. Where regulatory systems provide stability and predictability, they enable capital accumulation, entrepreneurial expansion, and sustained growth. Where they remain fragmented and inconsistent, they amplify uncertainty and limit development potential.

**Hypotheses**

**Central Hypothesis (H _0_)**

Differences in regulatory efficiency—reflected in administrative costs, procedural complexity, and overall business-environment quality—are systematically associated with differences in GDP per capita growth between China and selected Latin American economies, with China’s more efficient regulatory framework linked to superior growth performance.

H1. Higher administrative burdens—measured by greater costs of construction permits and a higher number of property registration procedures—are negatively associated with GDP per capita growth, controlling for inflation, trade openness, gross capital formation, and rule of law.

H2. Higher regulatory quality—captured by ease-of-doing-business scores and property registration scores—is positively associated with GDP per capita growth, reflecting the growth-enhancing effects of reduced transaction costs and greater institutional predictability.

H3. After controlling for macroeconomic and institutional factors, Latin American countries exhibit systematically lower growth performance relative to China, indicating that China’s regulatory efficiency and institutional coherence account for a significant share of the observed growth differential.

## 3. Method

This study uses a panel data approach to analyze the impact of regulatory efficiency and institutional quality on economic growth. The dataset covers seven countries—Argentina, Brazil, Chile, Colombia, Mexico, Peru, and China—over the period 2003–2019. The five Doing Business indicators—costs for construction permits, construction permit scores, ease of doing business, property registration score, and number of property registration procedures—represent the broader variable of regulatory efficiency, capturing administrative burdens, procedural complexity, and the overall conduciveness of the business environment.

Panel data models allow control for unobserved heterogeneity across countries (country-specific characteristics) and over time (temporal shocks), providing more accurate estimates of the effect of regulatory and institutional variables on GDP growth. Both fixed-effects (FE) and random-effects (RE) specifications are estimated. Model selection is guided by the Hausman test, ensuring appropriate handling of correlation between regressors and unobserved effects.

### 3.1 Econometric model

**Dependent variable:**

GDP growth_it = Annual GDP growth for country i in year t.

**Treatment variable:**

Regulatory Efficiency_it = The 5 variables of Doing Business.

**Control variables:**

• Trade openness (% of GDP): captures benefits of international integration and technology diffusion.
• Inflation (annual %): reflects macroeconomic stability; high inflation is expected to negatively impact growth.
• Gross capital formation (annual %): represents domestic investment in productive capacity.
• Rule of Law (percentile rank): proxies institutional quality and enforcement of contracts and property rights.

The fixed-effects model accounts for time-invariant country characteristics, denoted as α_i. In this specification, i = 1, …,7 represents the countries in the sample, t = 2003, …,2019 represents the time period, and u_it is the idiosyncratic error term. By using this approach, the model isolates the effect of within-country variation over time, effectively controlling for all time-invariant factors that might otherwise bias the estimates.

The random-effects model assumes that the country-specific effects, denoted as μ_i, are uncorrelated with the regressors. These effects are modeled as normally distributed with mean zero and variance σ_μ ^2^, while the idiosyncratic error term u_it is also normally distributed with mean zero and variance σ_u ^2^. The μ_i term captures unobserved heterogeneity across countries. When this assumption of no correlation holds, the random-effects model is more efficient than the fixed-effects model, providing more precise estimates:

**Hausman test**

In this context, *β̂{FE} and β̂*{RE} represent the vectors of estimated coefficients from the fixed-effects and random-effects models, respectively, and *k* denotes the number of regressors. The Hausman test evaluates the null hypothesis ( *H _0_*) that the random-effects estimator is consistent, which requires that the country-specific effects ( *μ_i*) are uncorrelated with the regressors. A p-value greater than 0.05 indicates that the random-effects model is preferred, while a p-value below 0.05 favors the fixed-effects specification. This test ensures that the selected model produces unbiased and consistent coefficient estimates.

### 3.2 Sources and causal identification

The analysis uses data from the World Bank’s WDI and WGI (2003–2019), including GDP growth, inflation, trade openness, gross capital formation, and Rule of Law. Regulatory indicators—construction permit costs and scores, ease of doing business, and property registration procedures—come from Doing Business ( Table 2). These standardized sources ensure consistent, comparable measures across the seven countries.

**Table 2.** Variables, definitions, sources, and construction.

Variable	Definition	Source	Years
GDP growth (annual %)	Income level in current dollars	WDI	2003–2019
Inflation (%)	Annual consumer price inflation	WDI	2003–2019
Trade (% of GDP)	Trade openness index	WDI	2003–2019
Gross capital formation (growth %)	Investment growth	WDI	2003–2019
Rule of Law (percentile rank)	Institutional quality	WGI	2003–2019
Costs for construction permits	Cost of obtaining a construction permit	Doing Business	2003–2019
Costs for construction permits - Score	Scaled score reflecting the relative cost of construction permits	Doing Business	2003–2019
Ease of Doing Business	Overall score measuring how easy it is to do business in a country	Doing Business	2003–2019
Registering property procedures - Score	Scaled score representing the number of steps required to register property	Doing Business	2003–2019
Registering property procedures (number)	Actual number of steps needed to register property	Doing Business	2003–2019

Notes. All variables come from the World Bank’s WDI and WGI databases, except *Time to start a business*, taken from *Doing Business* (available only for 2003–2019). GDP per capita and its growth, inflation, trade openness, gross capital formation, and Rule of Law follow standard World Bank definitions. *Log GDP per capita (2015)* is the natural log of each country’s 2015 value. The start-up days variable is used only for descriptive purposes. Source: World Bank.

Causal inference is ensured through several strategies. The treatment variable—days required to start a business—is lagged by one year to reduce simultaneity bias. Fixed-effects models control for unobserved, time-invariant country characteristics, while standard errors are clustered at the country level to address heteroskedasticity and within-country correlation. Counts of Doing Business reforms are used as quasi-exogenous shocks to regulatory quality, strengthening identification.

## 4. Results

The analysis reveals clear structural differences between China and selected Latin American countries. Descriptive statistics and figures show that China consistently achieves higher GDP growth, lower inflation volatility, stronger governance, and more efficient regulatory processes, including lower costs and fewer procedures for business registration and property formalization.

Panel-data regressions demonstrate that these regulatory and institutional advantages are significantly linked to stronger per capita growth, with lower administrative burdens and higher ease-of-doing-business scores positively affecting outcomes across both fixed- and random-effects models. Hausman tests confirm the appropriate model choice, while dummy-variable regressions benchmark China against Latin America, showing that Latin American countries systematically experience lower growth relative to China.

### 4.1. Descriptive analysis

Table 3 presents key macroeconomic and institutional indicators for selected Latin American countries and China for the period 2003–2019. The indicators include annual GDP growth, inflation rates, trade openness (as a percentage of GDP), gross capital formation growth, and rule of law percentile ranks. These measures capture both economic performance and institutional quality, highlighting structural differences between China and Latin American economies.

**Table 3.** Descriptive statistics – All key variables (2003–2019).

Variable	Mean	Std. Dev.	Min	Max	Observations
GDP growth (annual %) overall	4.22	3.68	-6	14	N = 119
between	2.37	2.9	0	9.06	n = 7
within	2.95	−5.02	10.98		T = 17
Inflation (%) overall	4.71	6.09	−1	54	N = 119
between	15.19	2.71	44		n = 7
within	2.25	−5.29	14.71		T = 17
Trade (% of GDP) overall	46.19	15.44	22	81	N = 119
between	15.01	26.01	66.75		n = 7
within	6.62	32.21	63.51		T = 17
Rule of Law (percentile rank) overall	44.78	18.07	20	88	N = 119
Between	18.65	32.16	85.3		n = 7
Within	5.08	31.68	58.81		T = 17
Gross capital formation (growth %) overall	6.53	12.09	−25	40.2	N = 119
Between	3.38	2.14	11.7		n = 7
Within	11.67	−25.27	39.78		T = 17

**Note:** Table 3 reports key macroeconomic and institutional indicators for China and selected Latin American countries (2003–2019). China shows stronger governance, higher and more stable growth, lower inflation, and more predictable investment, while Latin America displays greater variability and weaker institutions. Data from the World Bank WDI and WGI.

The data shows that China consistently exhibits higher GDP growth, more stable macroeconomic conditions, and stronger governance indicators, while Latin American countries display greater variability and lower rule-of-law scores. The sources for these data are the World Bank’s World Development Indicators and the Worldwide Governance Indicators, which provide standardized, cross-country comparable measures for macroeconomic and institutional analysis.

**Figure 1.**
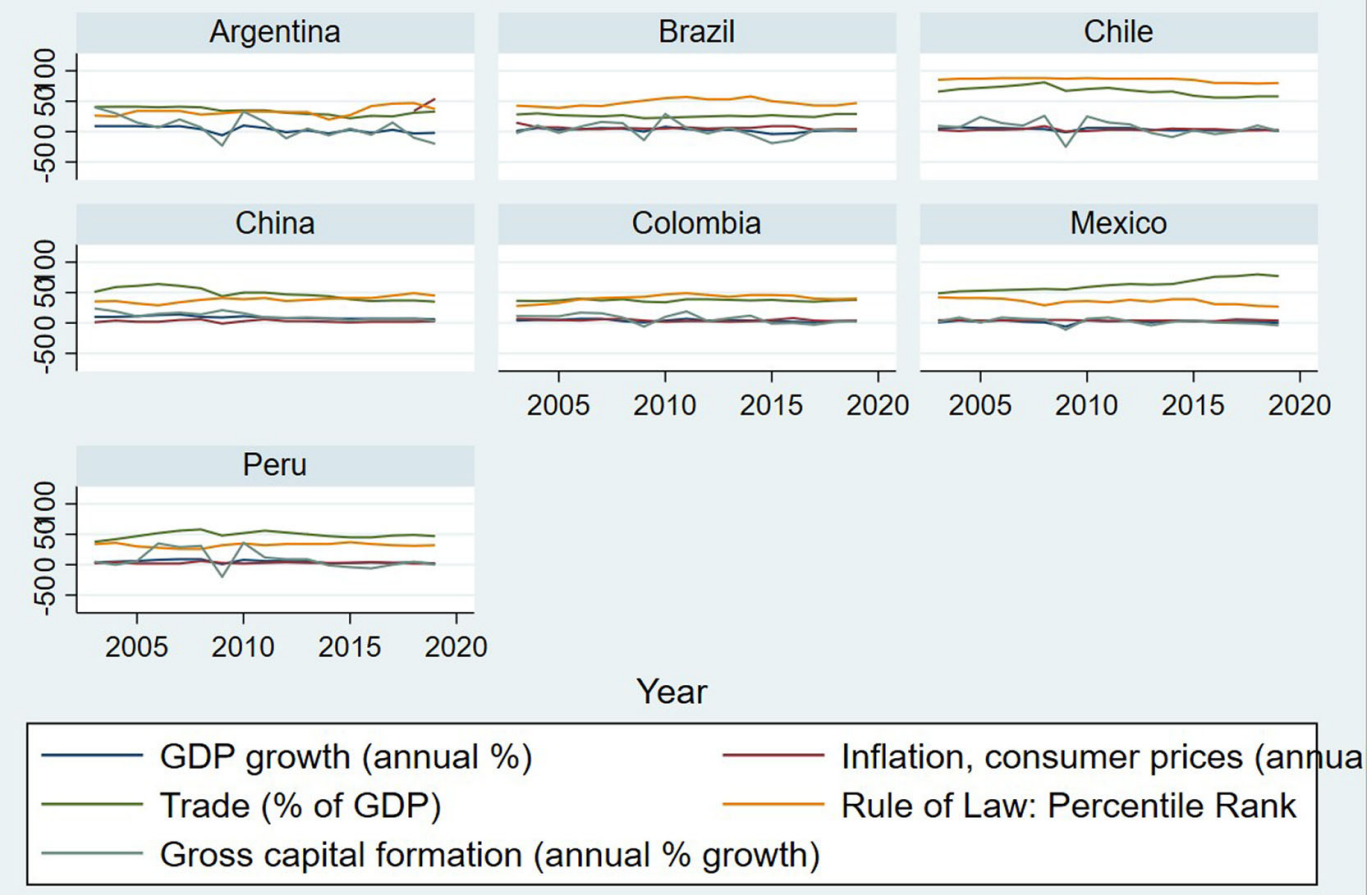
Graphic analysis of economic growth and control variables. Note: Visual comparison of macroeconomic performance and governance indicators. China exhibits stable growth and high rule-of-law scores, while Latin American countries display dispersed economic and institutional outcomes, emphasizing regional heterogeneity.

Figure 1 presents the analysis of GDP growth and macroeconomic controls. The figure illustrates that China exhibits strong growth alongside higher rule-of-law scores, lower inflation volatility, and more stable investment patterns, whereas Latin American countries show greater dispersion in economic performance and governance quality. This variation highlights the structural differences in institutional and macroeconomic environments.

Table 4 reports regulatory efficiency indicators, including costs and scores for construction permits, ease of doing business, and property registration procedures. China consistently demonstrates lower costs, higher scores, and fewer procedures, highlighting a more efficient business environment, whereas Latin American countries face higher costs, lower scores, and more administrative complexity. These contrasts establish China as a benchmark for regulatory efficiency and underscore the institutional gaps facing Latin American firms. *(See* Table 4 *).*

**Table 4.** Descriptive statistics of regulatory efficiency: construction permits, ease of doing business, and property registration procedures.

Variable	Mean	Std. Dev.	Min	Max	Observations
Costs for construction permits overall	4.49	3.48	0.8	11.8	N = 119
Between	3.71	1.14	10.21		n = 7
Within	1.22	0.37	7.47		T = 17
Costs for construction permit. Score overall	77.53	17.42	40.84	95.80	N = 119
Between	18.54	48.91	94.27		n = 7
Within	6.08	62.54	97.89		T = 19
Ease of Doing Business overall	65.32	5.95	55.53	73.55	N = 119
Between	6.41	55.53	72.03		n = 7
Within	1.13	63.00	66.85		T = 17
Registering property proce. - Score overall	49.73	17.48	0	78.75	N = 119
Between	22.55	0	70.12		n = 7
Within	5.18	39.32	60.15		T = 17
Registering property proc. (number) overall	7.08	2.24	3.55	13.61	N = 119
Between	2.92	4.59	13.61		n = 7
Within	0.62	5.83	8.33		T = 17

Note: Descriptive statistics on regulatory efficiency, including construction permit costs and scores, ease of doing business, and property registration procedures for China and selected Latin American countries. China consistently shows lower costs, higher scores, and fewer procedures, highlighting a more efficient regulatory environment, while Latin American countries face higher costs, lower scores, and more complex procedures. Data are primarily from the World Bank’s Doing Business database, complemented by the World Bank Enterprise Surveys.

**Figure 2.**
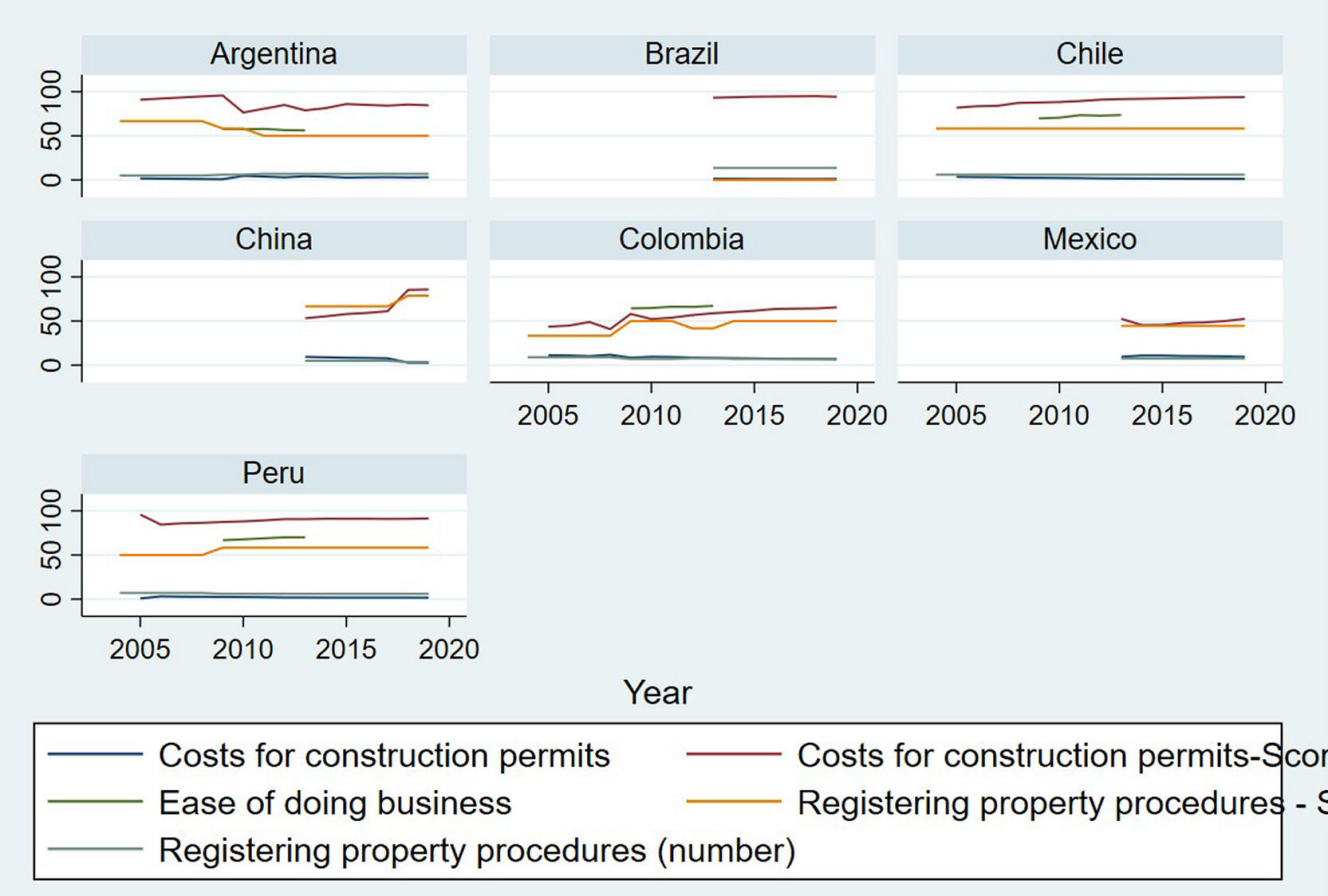
Graphic analysis of variables of regulatory efficiency. Note: Graphical analysis of regulatory efficiency indicators, including construction permit costs and scores, ease of doing business, and property registration procedures. China serves as a benchmark with minimal procedural delays, lower costs, and higher scores, whereas Latin American countries face higher administrative burdens, more costly procedures, and lower regulatory efficiency. Data are primarily from the World Bank’s Doing Business database, complemented by the World Bank Enterprise Surveys.

Figure 2 illustrates these regulatory indicators. China emerges as a clear benchmark with minimal procedural delays, lower costs, and higher scores across all measures. Latin American countries display higher administrative burdens, more costly procedures, and lower scores, confirming a persistent efficiency gap. These descriptive patterns form the basis for subsequent econometric analysis linking regulatory efficiency to economic performance.

### 4.2. Regression results

To assess the impact of regulatory efficiency on economic performance, we conducted panel-data regressions covering China and selected Latin American countries. Both fixed-effects (FE) and random-effects (RE) models were used to account for country-specific and temporal heterogeneity. The regression analysis focuses on key indicators of regulatory efficiency: construction permit costs, construction permit scores, ease of doing business, and property registration procedures (score and number of steps). These variables reflect administrative burdens, procedural complexity, and the overall conduciveness of the business environment.

The results, summarized in Table 5, indicate that higher regulatory efficiency is consistently associated with stronger GDP growth. In the FE specifications, lower construction permit costs and higher ease-of-doing-business scores positively correlate with growth, while in the RE specifications, fewer property registration procedures are linked to better economic performance. China consistently displays lower administrative burdens, higher regulatory scores, and fewer required procedures, which positions it as a benchmark for an efficient regulatory environment compared with the Latin American countries in the sample.

**Table 5.** GDP growth and regulatory environment: fixed effects (FE) and random effects (RE) specifications.

Variable	FE: Costs for construction permits (1)	FE: Costs for construction permits Score (2)	FE: Ease of doing business (3)	RE: Registering property proce. – Score (4)	RE: Registering property proc. (number) (5)
Regulatory Efficiency	0.1585 ** (0.062)	−0.0312 ** (0.012)	0.4743 ** (0.153)	0.0587 *** (0.013)	−0.456 *** (0.106)
Inflation (annual %)	0.0001 (0.109)	0.0005 (0.109)	0.048 (0.230)	−0.0810 *** (0.015)	−0.082 *** (0.016)
Trade (% of GDP)	0.0185 (0.021)	0.0187 (0.021)	0.102 *** (0.019)	−0.0308 ** (0.015)	−0.0318 ** (0.016)
Rule of Law (Percentile)	0.0311 (0.021)	0.0311 (0.021)	0.173 *** (0.040)	−0.0113 *** (0.0039)	−0.011 *** (0.0040)
Gross Capital Formation (annual %)	0.1536 *** (0.018)	0.1536 *** (0.018)	0.1163 *** (0.012)	0.1746 *** (0.016)	0.1740 *** (0.016)
Constant	−0.316 (0.573)	2.803 ** (1.085)	−42.919 ** (12.566)	2.491 *** (0.288)	8.656 *** (1.709)
N	119	119	119	119	119
Groups	7	7	7	7	7

Note: Regression results using fixed-effects (FE) and random-effects (RE) panel models. Variables include regulatory efficiency indicators (construction permits, procedures, ease of doing business), inflation, trade openness, gross capital formation, and Rule of Law percentile. Significance levels:

*** p < 0.01,

** p < 0.05,

* p < 0.1,

+ marginal p ≈ 0.05–0.1. Source: World Bank Enterprise Surveys, 2015–2023; World Bank WDI and WGI.

Macroeconomic control variables behave as expected. Gross capital formation contributes positively to GDP growth across all models, while inflation shows a generally negative effect. Trade openness presents mixed results but is significant in certain specifications. Rule of Law percentile ranks generally have a positive or modest effect, highlighting the relevance of institutional quality alongside regulatory efficiency.

To determine the appropriate panel-data specification, we conducted a series of Hausman tests comparing FE and RE models across different model specifications. The Hausman test evaluates whether the RE estimator is consistent by testing the null hypothesis that the individual effects are uncorrelated with the regressors.

As shown in Table 6, Tests 1 and 2 yield Chi ^2^ values of 2.30 and 2.31, with p-values exceeding 0.80, indicating no evidence to reject the null hypothesis. These results suggest that, for these specifications, the random-effects estimator is both consistent and efficient. Test 3, with a Chi ^2^ of 11.02 and a marginal p-value of approximately 0.05, suggests caution: while the RE model may still be acceptable, fixed effects could also be considered depending on robustness checks. In contrast, Tests 4 and 5 produce highly significant results (Chi ^2^ = 24.19 and 23.78, p < 0.001), leading to rejection of the null hypothesis and indicating that fixed-effects models are required for these specifications.

**Table 6.** Hausman test results for panel-data model selection.

Test	Chi ^2^ (Hausman)	Prob > Chi ^2^	Recommended model	Notes
1	2.30	0.8058	Random effects	p > 0.05 → cannot reject Ho, RE is consistent & efficient
2	2.31	0.8045	Random effects	p > 0.05 → same as above
3	11.02	0.0510	Random effects (cautious)	borderline p ≈ 0.05; RE may be acceptable
4	24.19	0.0002	Fixed effects	p < 0.05 → reject Ho, FE needed
5	23.78	0.0002	Fixed effects	p < 0.05 → reject Ho, FE needed

Note: The Hausman test checks if random-effects are consistent. Tests 1–3 suggest RE may be adequate; Tests 4–5 indicate FE is required. Data include GDP per capita growth, regulatory efficiency (permits, ease of doing business, property registration), macro controls (trade, inflation, capital formation), and Rule of Law for China and selected Latin American countries, from World Bank Enterprise Surveys (2015–2023), WDI, WGI, and Ease of Doing Business.

These results highlight the heterogeneity of the data structure across models and confirm that model selection must be guided by both statistical tests and theoretical considerations. For most specifications with high p-values, RE is adequate, but for those with significant Hausman tests, FE better accounts for correlation between the individual effects and regressors, ensuring unbiased and consistent coefficient estimates.

To further assess the differential impact of regulatory and institutional conditions on economic performance, we conducted a panel-data analysis using dummy variables to distinguish China from selected Latin American countries. Table 7 reports the results across four specifications: three random-effects models (columns 1–3) and one fixed-effects model (column 4). The dummy variables are coded as 1 for Latin American countries and 0 for China, using China as a benchmark for efficient regulatory practices and institutional quality.

**Table 7.** Impact of regulatory, institutional, and dummy variables on GDP growth.

Variable	Dummy *costs for construction permits (RE)	Dummy * costs for construction permits score (RE)	Dummy * ease of doing business (RE)	Dummy *regist. property proce. – score (FE)
**Gross capital formation**	0.262 ***	0.262 ***	0.253 ***	0.175 **
**Trade/GDP**	–	–	−0.130 ***	0.081 **
**Rule of law percentile**	–	–	0.058 ***	0.026
**Inflation**	–	–	–	−0.015
**Dummy**	–	−3.658 *	- 27.82 ***	–
**Dummy** * **regulatory efficiency**	−0.178 *	0.036	0.397	−0.004
**Constant**	3.234 ***	3.316 ***	8.258 ***	−2.030
**R** ^**2**^ **Overall**	0.647	0.647	0.855	0.348
**Observations**	119	119		71
**Groups**	7	7	7	7

Note: Models 1–3 use random effects, Model 4 uses fixed effects. Significance:

*** p < 0.01,

** p < 0.05,

* p < 0.1,

+ marginal p ≈ 0.05–0.1. Missing (−) indicates excluded variables. Diagnostics confirm panel robustness. Dummies: 1 = Latin America, 0 = China (benchmark). Data: World Bank Enterprise Surveys (2015–2023), WDI, WGI, Ease of Doing Business.

The results in Table 7 show that China consistently achieves more favorable growth outcomes relative to Latin America. Coefficients for the Latin American dummies are generally negative or smaller than the baseline (China), with column 3 reaching statistical significance at the 1% level (−27.82), underscoring the substantial gap in growth performance linked to differences in regulatory efficiency, institutional quality, and governance structures.

Control variables behave as expected: gross capital formation remains positively and strongly associated with growth, trade and Rule of Law show mixed but coherent effects, and inflation has a modest negative effect in the FE specification. Overall, these findings confirm that China serves as a practical benchmark in the region, with lower administrative burdens, more predictable regulatory processes, and stronger governance translating into more stable and higher per capita growth compared to Latin America.

## 5. Discussions

The empirical results align with and deepen existing debates on institutions and development. In line with Hamner (2002), who underscored the competitiveness gains associated with China’s gradual market opening, the evidence here indicates that what matters most is not liberalization in isolation but the administrative and regulatory conditions under which markets operate. Lower procedural complexity, reduced compliance costs, and stronger business-environment scores are consistently associated with higher GDP per capita growth. Conversely, the heavier administrative burdens observed in Latin America are not neutral frictions; they are systematically correlated with weaker macroeconomic performance. Regulatory inefficiency, therefore, carries tangible growth costs.

The findings also challenge the convergence argument advanced by Hearn and León-Manríquez (2011). Although both regions experienced post-2009 institutional adjustments, the data do not support sustained institutional alignment. Instead, panel estimations and benchmark regressions point to persistent divergence. China’s regulatory consolidation—particularly the strengthening of enforcement and institutional coordination noted in Valdiglesias (2026; 2025)—appears to have enhanced predictability and policy coherence. In contrast, Latin American reforms remain uneven and frequently undermined by fragmented implementation. This pattern is consistent with evidence of structural weaknesses in corporate governance and SME productivity documented by Chong and Lopez de Silanes (2007) and Cardoza et al. (2016). Even after accounting for inflation, trade openness, capital formation, and rule of law, administrative delays and procedural burdens retain a statistically significant association with lower growth.

Sector-specific research reinforces this macro-level divergence. Persistent weaknesses in infrastructure and electricity oversight in Latin America ( Suárez-Alemán et al., 2019; Ravillard et al., 2010; Andres, 2014), together with broader institutional fragility affecting SMEs ( Chong and Lopez de Silanes, 2007; Valdiglesias, 2024; McKay et al., 2018; Vassolo et al., 2012), illustrate how regulatory gaps translate into structural constraints. By contrast, East Asia’s integration of industrial policy, coordinated supervision, and infrastructure investment ( Shen et al., 2025; Sims et al., 2025; Kastner and Pearson, 2021; Kim and Dong, 2025; Weng and Li, 2024; Zhang et al., 2022) reduces uncertainty and supports long-term planning. Even when outward investment strategies are pragmatically oriented ( Dollar, 2017; Moran et al., 2012), domestic regulatory coherence remains a distinguishing advantage. Overall, the evidence supports the view that predictable, enforceable, and strategically aligned regulation is a structural determinant of divergent growth trajectories.

This study contributes to comparative political economy by providing systematic quantitative evidence that links regulatory efficiency to GDP per capita growth across regions. Through a panel-data design that incorporates fixed- and random-effects models with Hausman diagnostics, it moves beyond descriptive comparisons and estimates the magnitude of regulatory costs. By treating China as an empirical benchmark rather than a normative template, the analysis refines debates on institutional quality, state capacity, and transaction-cost efficiency.

For Latin America, the results point to clear institutional priorities. Simplifying business entry procedures, reducing construction and registration costs, and strengthening enforcement credibility are not merely technical adjustments but growth-enhancing reforms. The effectiveness of independent regulatory agencies depends on coordination capacity and insulation from fragmentation must occur alongside improvements in oversight and governance to prevent the inefficiencies observed in earlier privatization waves. Investments in infrastructure and digital regulatory systems can further reduce transaction costs and improve policy credibility.

Further research should investigate firm-level transmission channels through which regulatory simplification affects productivity, innovation, and investment decisions, particularly among SMEs. Comparative longitudinal analyses could clarify how regulatory reform interacts with industrial policy and digital governance. Such work would help refine understanding of how institutional design translates into sustained and inclusive economic growth across diverse political and regulatory contexts.

## Ethics and consent

Ethical approval and consent were not required.

## Data Availability

All datasets used in this study are publicly available. The datasets and processed data are cited below following the reference style used throughout the manuscript:
• World Development Indicators (WDI) • World Bank. *World Development Indicators.* Washington, DC: World Bank; 2026. Available at: https://databank.worldbank.org/source/world-development-indicators. Publicly accessible. Variables used: GDP growth, inflation, trade openness, gross capital formation. • Worldwide Governance Indicators (WGI) • World Bank. *Worldwide Governance Indicators.* Washington, DC: World Bank; 2026. Available at: https://info.worldbank.org/governance/wgi/Home/Reports. Publicly accessible. Variables used: rule of law, regulatory quality. • Enterprise Surveys • World Bank. *Enterprise Surveys.* Washington, DC: World Bank; 2026. Available at: https://www.enterprisesurveys.org/. Publicly accessible. Variables used: firm-level regulatory data. • Processed dataset for replication (Extended Data) • Valdiglesias J. *Processed dataset for replication of statistical analysis.* Zenodo; 2026. DOI: https://doi.org/10.5281/zenodo.18727744. ( Valdiglesias, J. 2026b) Licensed under **CC-BY 4.0**.

## References

[ref1] Abreo C Bustillo R Rodriguez C : The role of institutional quality in the international trade of a Latin American country: evidence from Colombian export performance. *J. Econ. Struct.* 2021;10(1):24. 10.1186/s40008-021-00253-5 34815926 PMC8603646

[ref2] Ahlstrom D Bruton GD Yeh KS : Private firms in China: Building legitimacy in an emerging economy. *J. World Bus.* 2008;43(4):385–399. 10.1016/j.jwb.2008.03.001

[ref3] Albright ZC : The Political and Pragmatic Determinants of Chinese Development Finance in Latin America and the Caribbean, 2008–2019. *Latin American Politics And Society.* 2025;67:78–97. 10.1017/lap.2024.54

[ref4] Andres L : *Assessing the governance of electricity regulatory agencies in Latin America.* World Bank Publications;2014. Reference Source

[ref5] Araujo JT Brueckner M Clavijo M : *Benchmarking the determinants of economic growth in Latin America and the Caribbean.* World Bank;2014. (Report No. 91015-LAC). Reference Source

[ref6] Bello MLA Villegaz MG Acuña OAE : Effects of corporatization on the financial performance of non-financial state-owned enterprises in Latin America between 1999 and 2018. *Rev Bras Polit Publicas.* 2023;12(3). 10.5102/rbpp.v12i3.8522

[ref7] Bezuidenhout H Mhonyera G Van Rensburg J : Emerging Market Global Players: The Case of Brazil, China and South Africa. *Sustainability.* 2021;13(21):12234. 10.3390/su132112234

[ref9] Cardoza G Fornes G Farber V : Barriers and public policies affecting the international expansion of Latin American SMEs: Evidence from Brazil, Colombia, and Peru. *J. Bus. Res.* 2016;69(6):2030–2039. 10.1016/j.jbusres.2015.10.148

[ref11] Chen L Kenjayeva U Mu G : Evaluating the influence of environmental regulations on green economic growth in China: A focus on renewable energy and energy efficiency guidelines. *Energ. Strat. Rev.* 2024;56:101544. 10.1016/j.esr.2024.101544

[ref12] Chong A Lopez de Silanes F , editors. *Privatization in Latin America: Myths and reality.* World Bank;2005. Reference Source

[ref13] Chong A López de Silanes F : *Investor protection and corporate governance: Firm-level evidence across Latin America.* The Inter-American Development Bank;2007. Reference Source

[ref14] De Barrios MLC Domínguez EML Treviño J o M : Latin America and China: international trade and economic growth. *Anal Econ.* 2023;38(99):23–52. 10.24275/uam/azc/dcsh/ae/2023v38n99/cardozo

[ref16] Dollar D : China’s investment in Latin America. *GEOECONOMICS AND GLOBAL ISSUES PAPER 4.* 2017. Reference Source

[ref17] Fornes G Mendez A , editors. *The China-Latin America Axis: Emerging Markets and their Role in an Evolving Global Economy.* Palgrave;2018. Reference Source

[ref19] Freites A : Factors determining the emergence of environmental concerns regarding Chinese investment in the South America’s extractive industries. *The Extractive Industries And Society.* 2024;19:101506. 10.1016/j.exis.2024.101506

[ref20] Gaganis C Papadimitri P Pasiouras F : Informal institutions and corporate reputational exposure: The role of public environmental perceptions. *Br. J. Manag.* 2021;32(4):1027–1061. 10.1111/1467-8551.12461

[ref21] Girón A : Large Chinese corporations in Latin America: more extraction or a just transition to an environmentally sustainable economy?. *Edward Elgar Publishing eBooks.* 2025; pp.191–221. 10.4337/9781035340262.00021

[ref23] Gutierrez LH Berg S : Telecommunications liberalization and regulatory governance: Lessons from Latin America. *Telecommun. Policy.* 2000;24(10–11):865–884. 10.1016/S0308-5961(00)00069-0

[ref24] Hamner KJ : The globalization of law: International merger control and competition law in the United States, the European Union, Latin America, and China. *J Transnatl Law Policy.* 2002;11:385.

[ref25] Hearn AH León-Manríquez JL , editors. *China engages Latin America: Tracing the trajectory.* Lynne Rienner Publishers;2011. Reference Source

[ref26] Irwin A Gallagher KP : Chinese mining in Latin America: A comparative perspective. *Lat Am Polit Soc.* 2013;22(2):1–30. 10.1177/1070496513489983

[ref27] Jenkins R : *How China is reshaping the global economy: Development impacts in Africa and beyond.* Oxford University Press;2022. Reference Source

[ref28] Kastner SL Pearson MM : Exploring the parameters of China’s economic influence. *Stud. Comp. Int. Dev.* 2021;56(1):18–44. 10.1007/s12116-021-09318-9 33688107 PMC7934344

[ref29] Kim W Dong Y : China-Latin America Relations, 2000-2024: A Triangular Dynamic of Opportunities and Challenges. *México y la Cuenca del Pacífico.* 2025;14(41):9–37. 10.32870/mycp.v14i41.937

[ref31] Križić I : Regulating public procurement in Brazil, India, and China: Toward the regulatory-developmental state. *Regulation & Governance.* 2021;15(3):561–580. 10.1111/rego.12243

[ref32] Lei Y : China–Latin America relations in the context of the Belt and Road Initiative. *Dev. Policy Rev.* 2024;42(6). 10.1111/dpr.12814

[ref33] Lopez D Munoz F : China’s trade policy towards Latin America: an analysis of free trade agreements policy. *Asian Educ Dev Stud.* 2020;10(3):399–409. 10.1108/aeds-08-2019-0133

[ref34] Mayer F Gereffi G : Regulation and Economic Globalization: Prospects and Limits of Private Governance. *Bus. Polit.* 2010;12(3):1–25. 10.2202/1469-3569.1325

[ref35] Mazé D Alcaraz J Buitrago R RE : Emerging market multinationals’ embeddedness in Global South countries: an empirical study of Chinese MNEs in Peru. *Crit. Perspect. Int. Bus.* 2024;20(4):517–538. 10.1108/cpoib-09-2023-0087

[ref36] McKay BM Alonso-Fradejas A Brent ZW : China and Latin America: Towards a new consensus of resource control?. *Rural transformations and agro-food systems.* Routledge; 1st ed. 2018; pp.20. 10.4324/9781351008686

[ref37] Mechelli A Cimini R : The effect of corporate governance and investor protection environments on the value relevance of new accounting standards: the case of IFRS 9 and IAS 39. *J. Manag. Gov.* 2020;25(4):1241–1266. 10.1007/s10997-020-09551-9

[ref38] Mihut G Cullinan J Flannery D : International student mobility, Covid-19, and the labour market: a scoping review. *Comp. Migr. Stud.* 2025;13(1). 10.1186/s40878-025-00426-2

[ref39] Moran T Kotschwar BR Muir J : *Chinese investment in Latin American resources: The good, the bad, and the ugly (Peterson Institute for International Economics Working Paper No. 12-3).* Peterson Institute for International Economics;2012. Reference Source

[ref41] Nazmi N Revilla JE : *Economic efficiency and growth: Evidence from Brazil, China and India (WIDER Research Paper No. 2008/86).* UNU-WIDER;2008. Reference Source

[ref42] North DC : *Institutions, institutional change and economic performance.* Cambridge University Press;1990. 10.1017/CBO9780511808678

[ref43] OECD Development Centre: *The visible hand of China in Latin America.* OECD Development Centre;2015. Reference Source

[ref44] Ravillard P Carvajal F Lopez Soto DD : *Towards greater energy efficiency in Latin America and the Caribbean.* Inter-American Development Bank;2010. Reference Source

[ref46] Reyes JIR : Factores determinantes del desempeño empresarial en Lima Metropolitana durante la pandemia del COVID-19. *Quipukamayoc.* 2021;29(61):95–104. 10.15381/quipu.v29i61.21731

[ref47] Rocha HVP : Foreign Direct Investment, Structural Change, and Labor Allocation: Comparative Evidence from Asia and Latin America. *J. Econ. Integr.* 2025;40. 10.11130/jei.2024050

[ref50] Rousham E Clark M Latham M : Resilience and vulnerabilities of urban food environments in the Asia-Pacific region. *Matern. Child Nutr.* 2023. 10.1111/mcn.13513 PMC12647982 37097115

[ref51] Scott WR : *Institutions and organizations: Ideas and interests.* Sage Publications; 3rd ed. 2008. Reference Source

[ref52] Shen Y Yang H Zhu Q : How does the institutional environment improve the entrepreneurial quality of returnees? A configuration analysis based on a complex system view. *PLoS ONE.* 2025;20(7):e0322863. 10.1371/journal.pone.0322863 40591559 PMC12212558

[ref53] Sims JP Lee Y Lee BTF : New Chinese Economic Policy to Latin America? A QCA Approach to the Belt and Road Initiative. *Chin. Political Sci. Rev.* 2025;10. 10.1007/s41111-023-00244-w

[ref54] Soto GH Martinez-Cobas X : The impact of transportation investment, road transportation and telecommunications on FDI in Latin America 2008-2021. *Transport Economics And Management.* 2024;2:45–57. 10.1016/j.team.2024.01.002

[ref55] Stallings B : Changing international hegemony and dependency in peripheral countries: A case study of Latin America. *Compet. Chang.* 2024;29(3–4):315–333. 10.20542/0131-2227-2025-69-4-83-96

[ref56] Suárez-Alemán A Serebrisky T Perelman S : Benchmarking economic infrastructure efficiency: How does the Latin America and Caribbean region compare?. *Util. Policy.* 2019;58:1–15. 10.1016/j.jup.2019.03.003

[ref57] The world Bank: Formal Sector Enterprise Surveys. 2025. Reference Source

[ref59] Urrego-Sandoval C Pardo RP : Asia and Latin America Relations in the Twenty-First Century: A Review. *Colomb Int.* 2023;113:3–21. 10.7440/colombiaint113.2023.01

[ref60] Valdiglesias J : From a rent-seeking economy toward an entrepreneurial economy in Latin America: The role of institutional quality reform. From a rent-seeking economy toward an entrepreneurial economy in Latin America.pdf. 2012.

[ref61] Valdiglesias J : Analyzing the state institutions on the Latin American economic development from the East Asian evidences. *Peruvian Journal of Asia-Pacific Studies.* 2018;2(1):181–192. Reference Source

[ref62] Valdiglesias J : El crecimiento de China y la trampa del ingreso medio en Latinoamérica. *Rev IECOS.* 2024;25(1):94–110. 10.21754/iecos.v25i1.2119.

[ref63] Valdiglesias J : From governance to growth: Comparative insights on institutional reforms in China and Latin America. *Frontiers in Political Science.* 2025;7. 10.3389/fpos.2025.1718532

[ref64] Valdiglesias J : Beyond discipline: How Xi Jinping’s anticorruption policy triggered structural change in China. *Public Administration in Authoritarian Regimes* ;2026;82–104. Reference Source

[ref65] Valdiglesias J : Dataset&outout. *Zenodo.* 2026b. 10.5281/zenodo.18727744

[ref66] Vassolo RS De Castro JO Gomez-Mejia LR : Managing in Latin America: Common issues and a research agenda. *Acad. Manag. Perspect.* 2012;25(4):1–19. 10.5465/amp.2011.0129.

[ref67] Vieira VGR Vadlamannati KC Li Y : Dispositional balancing and hegemonic order: US response to China’s financial statecraft. *The Chinese Journal of International Politics.* 2023;16(1):1–30. 10.1093/cjip/poac025

[ref68] Vorotnikova T : What Does It Seek in a Far Country? Factors of Latin American Countries’ Interest in BRICS. *World Econ Int Relat.* 2025;69(4):83–96. 10.20542/0131-2227-2025-69-4-83-96

[ref69] Vrontis D Shams R Thrassou A : Global strategy evolution, devolution or revolution: Disruptions to globalization and international business introversion. *J. Int. Manag.* 2024;30(5):101188. 10.1016/j.intman.2024.101188

[ref70] Walsh PR Ferro M : Developing a framework for sustainable development in extractive industries: a Latin America perspective. *International Journal of Innovation and Sustainable Development.* 2020;14(1):67. 10.1504/ijisd.2020.104242

[ref71] Wang Z Feng K : The inclusiveness of China’s development finance: China’s hybrid approach to aid and poverty reduction in Africa. *Elementa Science of The Anthropocene.* 2023;11(1). 10.1525/elementa.2022.00066

[ref72] Weng Y Li W : Navigating Global Economic Dynamics: Institutional Innovation and Rule of Law in China’s Pilot Free Trade Zones. *J. Knowl. Econ.* 2024. 10.1007/s13132-024-01783-6

[ref73] Williamson OE : *Markets and hierarchies: Analysis and antitrust implications.* Free Press;1975. 10.1093/oxfordhb/9780199646135.013.12

[ref74] Williamson OE : Transaction-cost economics: The governance of contractual relations. *J. Law Econ.* 1979;22(2):233–261. 10.1086/466942 Reference Source

[ref75] Williamson OE : *The economic institutions of capitalism: Firms, markets, relational contracting.* Free Press;1985. 10.2307/1960952

[ref76] Wise C : China and Latin America's emerging economies: New realities amid old challenges. *Lat Am Policy.* 2016;7(1). 10.1111/lamp.12087

[ref77] Wise C Ching VC : Conceptualizing China–Latin America relations in the twenty-first century: The boom, the bust, and the aftermath. *Third World Q.* 2017;38(3):553–572. 10.1080/09512748.2017.1408675.

[ref78] Wise C Quiliconi C : China’s surge in Latin American markets: Policy challenges and responses. *Policy Polit.* 2007;35(3):410–438. 10.1111/j.1747-1346.2007.00067.x

[ref80] Zambrano-Monserrate MA : The role of governance in large-scale mining sector in Latin America. *Environ. Sci. Pol.* 2025;168:104071. 10.1016/j.envsci.2025.104071

[ref81] Zhang Y Wang J Chen J : Does environmental regulation policy help improve business performance of manufacturing enterprises? evidence from China. *Environ. Dev. Sustain.* 2022;25(5):4335–4364. 10.1007/s10668-022-02245-2

[ref82] Zhou J : A double-edged sword: Chinese direct investment in Latin America. *Struct. Chang. Econ. Dyn.* 2023;67:234–249. 10.1016/j.strueco.2023.07.010

