# Supplementary material for: Regulatory Design and Long-Term Growth: Insights from China’s Experience for Latin America
Source: F1000Res. 2026 Jun 9;15:469. Originally published 2026 Apr 2. [Version 2] doi: 10.12688/f1000research.178466.2 (PMC13284606; doi:10.12688/f1000research.178466.2)
Supplement: Peer review history

# Reviewer report: Reviewer response for version 2

**DOI:** 10.5256/f1000research.202300.r492330

**Published:** 2026-06-22

**Authors:** Ning Leng

**Affiliations:** 1 Georgetown University, Washington, District of Columbia, USA

The revisions incorporated both reviewers' suggestions and are satisfactory. I recommend indexing.

Is the work clearly and accurately presented and does it cite the current literature?

Partly

If applicable, is the statistical analysis and its interpretation appropriate?

Not applicable

Are all the source data underlying the results available to ensure full reproducibility?

Yes

Is the study design appropriate and is the work technically sound?

Yes

Are the conclusions drawn adequately supported by the results?

Partly

Are sufficient details of methods and analysis provided to allow replication by others?

Yes

Reviewer Expertise:

Political economy, comparative politcs, state-business relations, China, Global China.

I confirm that I have read this submission and believe that I have an appropriate level of expertise to confirm that it is of an acceptable scientific standard.

# Reviewer report: Reviewer response for version 2

**DOI:** 10.5256/f1000research.202300.r492329

**Published:** 2026-06-19

**Authors:** Eduardo Tzili Apango

**Affiliations:** 1 Universidad Autonoma Metropolitana, Mexico City, Mexico City, Mexico

The revised manuscript has satisfactorily addressed the major concerns raised during peer review. Although a few methodological issues remain only partially resolved and could be further refined in future iterations, the revisions substantially strengthen the theoretical balance, empirical interpretation, and transparency of the study. I therefore consider that the article meets the standards required for indexing and recommend its approval in its current form.

Is the work clearly and accurately presented and does it cite the current literature?

Partly

If applicable, is the statistical analysis and its interpretation appropriate?

Partly

Are all the source data underlying the results available to ensure full reproducibility?

Yes

Is the study design appropriate and is the work technically sound?

Partly

Are the conclusions drawn adequately supported by the results?

Partly

Are sufficient details of methods and analysis provided to allow replication by others?

Partly

Reviewer Expertise:

China Studies, International Relations, China-Latin America Relations.

I confirm that I have read this submission and believe that I have an appropriate level of expertise to confirm that it is of an acceptable scientific standard.

# Reviewer report: Reviewer response for version 1

**DOI:** 10.5256/f1000research.196855.r475912

**Published:** 2026-06-02

**Authors:** Eduardo Tzili Apango

**Affiliations:** 1 Universidad Autonoma Metropolitana, Mexico City, Mexico City, Mexico

The manuscript examines the relationship between regulatory efficiency and long-term economic growth through a comparative analysis of China and selected Latin American economies. The study draws on Institutional Theory and Transaction Cost Economics, and argues that coherent regulatory design, lower administrative burdens, and stronger enforcement capacity contribute to higher GDP growth. Using panel data for seven countries between 2003 and 2019, and estimating fixed-effects and random-effects models with standard macroeconomic controls, the article concludes that China’s regulatory environment constitutes a benchmark associated with superior economic performance and that Latin American economies face persistent institutional and administrative constraints that weaken growth outcomes. The manuscript addresses an important and policy-relevant question and demonstrates familiarity with recent literature on institutions, governance, and development. The empirical strategy and the effort to combine theoretical discussion with quantitative analysis represent clear strengths.

 At the same time, several aspects of the manuscript require revision before the study can be considered fully scientifically sound. Although the article is generally well presented and cites recent literature, the presentation occasionally overstates both the coherence of empirical evidence and the degree to which the findings support causal claims. The literature review is extensive and up to date, but at times it moves beyond establishing analytical context and adopts evaluative interpretations of China’s institutional performance that are not fully demonstrated by the empirical analysis. The manuscript would benefit from a clearer distinction between theoretical expectations, descriptive observations, and econometric findings. Several statements regarding China’s regulatory advantages should be reformulated more cautiously and grounded directly in the variables that are measured.

 The study design is appropriate for an exploratory comparative analysis. Nevertheless, important methodological limitations remain insufficiently addressed. The panel includes only seven countries and combines indicators originating from different databases and time horizons. More importantly, the empirical framework assumes that observed growth differences can be attributed to regulatory efficiency after controlling for a limited set of macroeconomic variables, but the possibility of omitted-variable bias remains substantial. Structural factors such as industrial composition, export specialization, demographic dynamics, state capacity, financial development, and technological upgrading are not incorporated and may partially explain the estimated differences. The manuscript should therefore moderate claims of explanatory sufficiency and explicitly acknowledge the limits of cross-country benchmarking.

 The methods and analytical procedures are described in broad terms, but do not yet provide sufficient detail for full replication. Although the author reports publicly available datasets, and provides a processed dataset repository, the manuscript should specify the exact construction of each variable, indicate how missing observations were handled, provide the complete model equations, clarify whether variables were transformed or standardized, explain the clustering procedure in greater detail, and document the implementation of lag structures. The discussion of quasi-exogenous regulatory shocks through Doing Business reforms is currently underdeveloped and should explain precisely how reform counts were operationalized and incorporated into estimation.

 Statistical analysis is generally appropriate for the research question, but aspects of interpretation require revision. The use of fixed-effects and random-effects models with Hausman diagnostics is methodologically acceptable. However, the interpretation of statistical significance occasionally exceeds what the results support. Several coefficients display mixed signs across specifications, and some institutional variables behave inconsistently. The manuscript should report robustness checks, discuss coefficient instability, and provide stronger justification for model selection where Hausman results are borderline. In addition, the language of causality should be replaced with more cautious formulations emphasizing association rather than demonstrated causal impact unless stronger identification strategies are introduced.

 The availability of source data is a positive feature of the article. Public datasets and replication datasets are reported, which substantially improves transparency and reproducibility.

 The conclusions are directionally consistent with the empirical results, but currently extend beyond what the evidence can support. The findings justify the argument that regulatory indicators are associated with economic performance and that China provides a useful comparative reference case. However, the article does not demonstrate that regulatory efficiency is the decisive mechanism explaining divergent growth trajectories. The conclusions should therefore be narrowed and reformulated to reflect the observational nature of the design.

Is the work clearly and accurately presented and does it cite the current literature?

Partly

If applicable, is the statistical analysis and its interpretation appropriate?

Partly

Are all the source data underlying the results available to ensure full reproducibility?

Yes

Is the study design appropriate and is the work technically sound?

Partly

Are the conclusions drawn adequately supported by the results?

Partly

Are sufficient details of methods and analysis provided to allow replication by others?

Partly

Reviewer Expertise:

China Studies, International Relations, China-Latin America Relations.

I confirm that I have read this submission and believe that I have an appropriate level of expertise to confirm that it is of an acceptable scientific standard, however I have significant reservations, as outlined above.

# Response

**Published:** 2026-06-03

**Authors:** Jhon Valdiglesias

**Affiliations:** UNMSM, Peru

Thank you for the detailed and constructive review. I have moderated the language throughout the manuscript to avoid strong causal interpretations and to clearly distinguish between theoretical expectations, descriptive evidence, and econometric results. The discussion of China has also been revised to ensure that it is presented strictly as a comparative empirical reference, without normative or overstated claims.

 In addition, the methodological section has been substantially expanded to improve transparency and replicability. This includes a clearer description of the construction of variables, the treatment of missing data, and the specification of the econometric models. I have also added more detail on the implementation of fixed and random effects models, as well as a clearer explanation of the Hausman test procedure and model selection.

 Furthermore, I have strengthened the discussion of limitations by explicitly acknowledging the risk of omitted variable bias and the constraints associated with the relatively small country sample. I believe the revised manuscript now provides a more balanced interpretation of the results, greater methodological clarity, and a clearer alignment between the empirical evidence and the conclusions.

# Reviewer report: Reviewer response for version 1

**DOI:** 10.5256/f1000research.196855.r475905

**Published:** 2026-05-19

**Authors:** Ning Leng

**Affiliations:** 1 Georgetown University, Washington, District of Columbia, USA

This article tackles a highly relevant and pressing question in comparative political economy: how regulatory efficiency and institutional design impact long-term economic growth, particularly when contrasting the divergent trajectories of China and Latin America. The author does a commendable job grounding the study in institutional and transaction cost theory, providing a clear theoretical logic for why predictable regulations lower transaction costs and foster development.

 The author has collected an impressive range of data, which I applaud. By deploying panel-data, the author tests the impact of specific regulatory bottlenecks. And to directly benchmark China against specific Latin American countries provides a novel perspective into the structural divergence between the two regions.

 While the manuscript provides a quantitative assessment of formal regulatory metrics, it could benefit from deeper nuance—specifically regarding the realities of informal politics and state capacity. Below I provide some main feedback for the author to consider.

 First, the primary limitation of the current manuscript is that it takes China's formal regulatory framework somewhat at face value, assuming a high degree of bureaucratic predictability and "disciplined state coordination". The author argues that in Latin America, "informal arrangements may temporarily compensate for weak formal governance" but ultimately "entrench opacity" and elevate transaction costs. However, the paper implicitly assumes that China has transcended these informalities through institutional consolidation. This dichotomy requires a more in-depth examination.

 China, much like Latin America, possesses a highly complex web of informal politics, discretionary regulation, and fragmented local governance. The "predictability" the author praises in China's licensing and property formalization often masks the reality that doing business in China relies heavily on dense, informal state-business networks to navigate shifting policy landscapes. Indeed, the author briefly acknowledges in the literature review that East Asian firms have historically navigated "weak property rights and shifting policy landscapes through dense state-business networks", but this crucial point is sidelined in the main analysis.

 The huge range of burdens on Chinese business and the highly unpredictable environment they face can be illustrated by works including my own book, “Politicizing Business: How Firms Are Made to Serve the Party-State in China” (Leng 2025),  “Governing and ruling: the political logic of taxation in China”(Zhang 2021) , “Capitalism Without Democracy: The Private Sector in Contemporary China” (Tsai 2007), “Between Developmental and Clientelist States: Local State-Business Relationships in China” (Ong 2012), “The Private Sector in Public Office: Selective Property Rights in China”,(Hou 2019), and many more.  

 Second, the manuscript highlights China's regulatory consolidation under Xi Jinping, specifically citing "reinforced anti-corruption enforcement" as a deliberate institutional recalibration that reduces rent-seeking and enhances coherence. However, the manuscript assumes the best regarding these initiatives. In practice, anti-corruption drives and regulatory crackdowns in authoritarian regimes are frequently discretionary, highly politicized, and selectively enforced against political rivals or specific sectors. By treating these structural shifts purely as "governance improvements", the article misses the fact that discretionary enforcement actually introduces a different, highly potent form of regulatory uncertainty for domestic and foreign actors, including companies. Some works that might help shed light on corruption and anti-corruption campaigns in China from different perspectives include “Double Paradox: Rapid Growth and Rising Corruption in China” (Wedeman 2012), “Taking China’s anticorruption campaign seriously”(Manion 2016), “Corruption and anti-corruption in reform China” (He 2000), “Corruption and anti-corruption in China: a review and future research agenda” (Tong 2022), and “Corruption stereotype and the unintended consequences of an anti-corruption campaign: evidence from the real estate sector in China” (Fang and Zhang 2025).

 Lastly, isolating the exact determinants of GDP growth remains inherently complex, and there is a risk that the current econometric specifications do not adequately control for all relevant factors. As highlighted by foundational economic literature, such as “I Just Ran Four Million Regressions” (Sala-i-Martin 1997), cross-country growth regressions are highly sensitive to model specification and context-specific interaction. While the author controls for inflation, trade openness, gross capital formation, and rule of law, China differs structurally from the Latin American countries across numerous other critical dimensions. Factors such as educational attainment, the depth of the financial system, labor market regulations, and domestic security conditions could be equally, if not more, consequential for growth. Although the study relies on Fixed-Effects and Random-Effects models to account for unobserved time-invariant heterogeneity, many of these crucial omitted variables change over time and possess highly interactive natures. Consequently, without explicitly controlling for them, the model risks overstating—or possibly understating—the true explanatory power of the regulatory variables.

 All in all, to strengthen the paper, the author could consider revise the theoretical framing and the discussion section to avoid a binary where China equals "formal predictability" and Latin America equals "informal fragmentation." Instead, the author should explore how informality operates *differently* in both regions. China's economic success may not be the result of a perfectly predictable, formal regulatory state, but rather a system where local discretionary politics and informal networks are temporarily aligned with growth and investment targets. Incorporating this political reality will make the comparison to Latin America significantly richer and more theoretically sound. I encourage the author to continue this important line of research.

Is the work clearly and accurately presented and does it cite the current literature?

Partly

If applicable, is the statistical analysis and its interpretation appropriate?

Not applicable

Are all the source data underlying the results available to ensure full reproducibility?

Yes

Is the study design appropriate and is the work technically sound?

Yes

Are the conclusions drawn adequately supported by the results?

Partly

Are sufficient details of methods and analysis provided to allow replication by others?

Yes

Reviewer Expertise:

Political economy, comparative politcs, state-business relations, China, Global China.

I confirm that I have read this submission and believe that I have an appropriate level of expertise to confirm that it is of an acceptable scientific standard, however I have significant reservations, as outlined above.

## References

[1] : Politicizing Business.2025; 10.1017/9781009662277 10.1017/9781009662277

[2] : Corruption stereotype and the unintended consequences of an anti-corruption campaign: evidence from the real estate sector in China. *Journal of Public Economics* .2025;249: 10.1016/j.jpubeco.2025.105474 10.1016/j.jpubeco.2025.105474

[3] : Corruption and anti-corruption in reform China. *Communist and Post-Communist Studies* .2000;33(2) : 10.1016/S0967-067X(00)00006-4 243-270 10.1016/S0967-067X(00)00006-4

[4] : The Private Sector in Public Office.2019; 10.1017/9781108632522 10.1017/9781108632522

[5] : Taking China’s anticorruption campaign seriously. *Economic and Political Studies* .2016;4(1) : 10.1080/20954816.2016.1152094 3-18 10.1080/20954816.2016.1152094

[6] : I Just Ran Four Million Regressions.1997; 10.3386/w6252 10.3386/w6252

[7] : Corruption andanti‐corruption in China: a review and future research agenda. *Asian-Pacific Economic Literature* .2022;36(1) : 10.1111/apel.12345 3-16 10.1111/apel.12345

# Response

**Published:** 2026-05-22

**Authors:** Jhon Valdiglesias

**Affiliations:** UNMSM, Peru

I thank the reviewer for the valuable comments and constructive suggestions. I have revised the manuscript to address the main concerns:

1. **Informality and China–Latin America comparison:**
  I now explicitly state that both regions are hybrid systems where formal and informal institutions coexist. The revised text avoids presenting China as fully formalized and instead emphasizes that informality operates differently across regions (more integrated in China’s state capacity, more fragmented in Latin America).
2. **Anti-corruption and regulatory consolidation:**
  I now clarify that anti-corruption campaigns in China may produce both governance improvements and regulatory uncertainty due to selective and discretionary enforcement.
3. **Methodological limitations:**
  I added explicit discussion of omitted variables and clarify that results should be interpreted as associations, not fully structural causal estimates.
